# Retinal Vein Occlusion after COVID-19 Vaccination—A Review

**DOI:** 10.3390/vaccines11081281

**Published:** 2023-07-26

**Authors:** Ho-Man Leung, Sunny Chi-Lik Au

**Affiliations:** 1 Hospital Authority, Ma Tau Wai 999077, Hong Kong; 2 Department of Ophthalmology, Tung Wah Eastern Hospital, So Kon Po 999077, Hong Kong

**Keywords:** COVID-19, COVID-19 vaccines, retinal artery occlusion, retinal vein occlusion, review

## Abstract

***Background*** Retinal vein occlusion (RVO) occurring after COVID-19 vaccination has been reported worldwide. Such a sight-threatening condition occurring after COVID-19 vaccination is a menace to ophthalmic health. This article reviews current evidence related to post-COVID-19 vaccination RVO. ***Method*** A total of 29 relevant articles identified on PubMed in January 2023 were selected for review. ***Observation*** All cases presented to ophthalmologists with visual loss shortly after COVID-19 vaccination. Mean and median age were both 58. No sex predominance was observed. RVO was diagnosed from findings on dilated fundal examination and ophthalmic imaging. AstraZeneca and BNT vaccines accounted for most cases. Vascular risk factors, e.g., diabetes mellitus and hypertension, were common. Most laboratory tests requested came back unremarkable. Most patients responded well to standard treatment, except those with ophthalmic comorbidities. Visual prognosis was excellent on short-term follow-up. ***Discussion*** The causality between RVO and COVID-19 vaccination is undeterminable because of the nature of articles, heterogenous reporting styles, contradicting laboratory findings and co-existing vascular risk factors. Vaccine-induced immune thrombotic thrombocytopenia, retinal vasculitis and homocysteinaemia were proposed to explain post-vaccination RVO. Large-scale studies have demonstrated that the incidence of RVO following COVID vaccination is very low. Nevertheless, the effects of boosters on retinal vasculature and ophthalmic health are still unclear. ***Conclusions*** The benefits of COVID-19 vaccination are believed to outweigh its ophthalmic risks. To ensure safe vaccination, the prior optimisation of comorbidities and post-vaccination monitoring are important. COVID-19 vaccines (including boosters) should be offered with reasonable confidence. Further studies are warranted to elucidate the ophthalmic impact of vaccines.

## 1. Introduction

COVID-19 vaccines have been widely delivered to fight against the COVID-19 pandemic. The health risks of these newly developed vaccines are hot research topics, especially regarding vascular endothelial complications. Several author groups have reported thromboembolic risks of COVID-19 vaccines [1,2,3], and thus ocular complications such as retinal vein occlusion (RVO) might be possible. With a high global coverage of vaccination [4], it is high time to review the literature regarding COVID-19-vaccine-related RVO.

## 2. Method

The literature was searched on PubMed on 15 January 2023 using (((vaccine) OR (vaccination) OR (after vaccination) OR (post vaccination)) AND ((COVID-19) OR (COVID) OR (SARS-CoV-2))) AND ((retinal vein occlusion) OR (retinal venous occlusion)). Titles and abstracts were screened.

A total of 37 articles were identified. After screening and review of reference lists of included articles, 29 relevant articles were included.

## 3. Clinical Characteristics of RVO following COVID-19 Vaccination

The age of patients ranged from 13 to 85. The median and mean of patients’ ages were both 58. The male to female ratio was around 1:1. These patients presented with visual diminution to ophthalmologists due to visual loss 15 min to 30 days after their last dose of vaccine. About 55% and 39% of cases occurred after the first dose and second dose, respectively. AstraZeneca and BNT vaccine-related RVO comprised most of the cases, while other types of COVID-19 vaccine, for example, Pfizer, Moderna and Covishield, were also reported.

Most patients had their intraocular pressure (IOP) documented. The IOP results, if reported, all fell within the normal range. The anterior segment and fundus were both checked as part of routine ophthalmic examination. No gross abnormalities, except cataracts, were reported on anterior segment examination. Typical fundal abnormalities of RVO on examination led to the diagnosis of RVO in most cases. Optical coherence tomography (OCT) and FA were performed for most of these patients to confirm the diagnosis. Optical coherence tomography angiography was performed in only a minority of cases.

Most centres performed an extensive laboratory work-up for their patients. Examples of abnormalities incidentally detected on work-up were as follows: Mildly reduced platelet count (129 × 10^9^/L) found in one patient with concurrent central retinal artery occlusion (CRAO) and central RVO (CRVO) [5]. One patient had mildly elevated glycated haemoglobin (6.7%) [6]. Raised d-dimer (547 ng/mL) was found in one case [7]. One patient had mildly raised inflammatory markers (erythrocyte sedimentation rate (ESR): 49, C-reactive protein (CRP): 14.6—unit not provided), rheumatoid factors (11, unit not provided) and d-dimer (6077.4 ng/mL) [8]. A mildly elevated homocysteine level was reported in two cases (16.4 and 22.19 micromol/L) [9,10]. Elevated lipid levels (total cholesterol: 227; LDL: 159, unit not provided) and mildly raised ESR (26, unit not provided) were detected from a case of combined CRAO and RVO [11]. Results of laboratory investigations for most other cases were unremarkable. Upon reviewing the past medical history of these cases, it was noted that a number of cases had background vascular risk factors, such as diabetes, hypertension, dyslipidaemia and atrial fibrillation.

The majority of the cases were treated with an intravitreal injection of anti-VEGF (vascular endothelial growth factor), an established treatment of RVO. Two other commonly used alternatives included intravitreal/intravenous corticosteroid and oral aspirin. Most patients reported an improvement in vision and fundal abnormalities resolved at follow-up visits. There were only few non-responders, and this was likely to be related to advanced age, concurrent retinal artery occlusion, which carries poor visual prognosis, and other comorbidities. As all these post-vaccination RVO cases occurred within these last 2 years, no results from long-term follow-up were provided from the case reports.

Summarised clinical information of individual cases can be found in Tables S1 and S2, attached in the Supplementary File. Table 1 presents the essence of clinical information from individual cases.

## 4. Discussion

Latest reviews and studies about post-vaccination RVO.

### 4.1. Recent Reviews on the Topic

Yeo et al. looked at the literature on post-vaccination retinal vascular occlusion. Their team noted that retinal vascular occlusion took place following the first dose in viral vector vaccines and following the second dose for mRNA vaccines [34]. Apart from RVO, retinal artery occlusion following COVID-vaccination has also been reported around the world [35,36]. Authors found the determination of causality between COVID-19 vaccination and retinal vascular events difficult [34,35,37].

### 4.2. Recent Relevant Studies Employing Big Data

It has come to our attention that there are two recently published retrospective cohort studies (with propensity score matching) investigating the incidence of retinal vascular occlusion following mRNA COVID-19 vaccination [38,39]. The authors of both studies (Dorney et al., Li et al.) performed statistical analyses on clinical data retrieved from electronic health records from the US. 

Dorney et al. stated that the incidence of newly diagnosed retinal vascular occlusion was 3.4 per 100,000 within 21 days of the first dose of mRNA COVID-19 vaccines [39]. No increased risk ratio with statistical significance was observed when compared to the recipients of influenza vaccines and tetanus, diphtheria and pertussis (Tdap) vaccines [39]. Dorney et al. compared the incidence of post-first-dose retinal vascular occlusion to that of post-second-dose, revealing a risk ratio of 2.25 (95% CI: 1.33–3.81) [39].

Li et al. found the hazard ratios of CRVO and branch RVO (BRVO) within 12 weeks of mRNA COVID-19 vaccination (compared to unvaccinated cohort) to be 3.97 (95% CI: 3.02–5.20) and 3.88 (95% CI: 3.02–4.97), respectively [38]. Li et al. also showed an increased risk of retinal vascular occlusion, as well as of all subtypes of retinal vascular occlusion in the vaccinated cohort at 2 years irrespective of sex, age and ethnicity [38].

There are a few points that are worth mentioning. First, the studies focused only on mRNA vaccines. The external validity may be affected, as the results may not be applicable to COVID-19 vaccines of other types [40]. Second, they used different cut-offs for defining vaccine-related retinal vascular occlusion. Li et al. also performed statistical analyses for retinal vascular occlusion that occurred within 2 years of vaccination. The differences in the endpoint selected may have had an influence on the statistical significance of results. On the other hand, whether late-onset RVO is directly related to COVID vaccines is a debatable issue. Thirdly, the populations used for comparison for the two studies were different. Li et al. used unvaccinated subjects for comparison, while Dorney et al. compared recipients of influenza and Tdap vaccines in the pre-COVID era. Lastly, the study by Li et al. placed more emphasis on the relative risks, while Dorney et al. pointed out that the absolute risk was small. The clinical importance of a statistically significant relative risk is equivocal if the absolute risk is very low. 

Hashimoto et al. investigated general ocular adverse effects of mRNA vaccines using big data, as well. They obtained data from a database based on a Japanese population [41]. This is one of the few large-scale studies that provide information on the ophthalmic side effects of vaccines in an Asian population. Hashimoto et al. detected a statistically significantly increased risk of retinal vein occlusion following the second dose of mRNA vaccines. Nonetheless, no increased risk of RVO and other ocular events after mRNA COVID vaccination was observed in their self-controlled case series study. The increased risk of RVO following the second dose was thought to be caused by residual confounding [41].

The caveats listed above are not exhaustive. Much caution should be exercised when interpreting studies of these kinds. That said, big data analyses are very promising in ophthalmology and vaccine side effect research. We look forward to studies looking at other types of vaccines and ophthalmic side effect surveillance with the use of big data.

## 5. Proposed Mechanisms of Suspected Vaccine-Related RVO

The exact mechanisms have yet to be ascertained. On review of the current evidence, three possible mechanisms were raised to explain this phenomenon. Little is known about whether the following mechanisms are independent or synergistic.

### 5.1. Vaccine-Induced Immune Thrombotic Thrombocytopenia

Chen Y et al. proposed that COVID-19 vaccines may be linked to several post-vaccination new-onset autoimmune conditions, one of which is vaccine-induced immune thrombotic thrombocytopenia (VITT) [42]. The anti-platelet factor 4 (PF4) antibody has been identified to be the culprit of this pathology [43]. The exact pathophysiology of this phenomenon is not certain, but it is believed that it resembles that of heparin-induced thrombocytopenia [44]. A few authors of the articles reviewed here have suggested that VITT may be implicated in vaccine-related RVO.

Nonetheless, the measurement of the anti-PF4 antibody level was only reported in five cases and no positive tests were reported [9,10,32]. That said, no conclusion on the effect of this phenomenon on post-vaccination RVO can be made based on the negative finding in just a few cases. To observe the etiological role of those antibodies in CRVO, it is recommended that the anti-PF4 level should be documented, if possible, in suspected cases of vaccine-related RVO in the future.

### 5.2. COVID-19 Vaccine as a Trigger in Homocysteinaemia

Parakh S et al. reported a case of RVO after COVID-19 vaccination with mildly elevated homocysteine (22.19 micromol/L) detected on investigation [9]. They attributed the mild homocysteinaemia to recent subclinical COVID-19 infection prior to vaccination (i.e., a very high level of SARS-CoV-2 IgG (>250 U/mL) 15 days after first dose) and suggested that COVID-19 vaccine may trigger venous thromboembolism in background homocysteinaemia (“two-hit hypothesis”). Parakh et al. also cited evidence of the potential effect of vaccines and their adjuvants on endothelial cells and autoimmunity. The theory of ischaemic pre-conditioning was quoted to support their two-hit hypothesis [45].

The validity of this hypothesis is questionable. Homocysteinaemia is known to be associated with arterial thromboembolism; even mild homocysteinaemia carries a risk of arterial thromboembolism [46]. Nonetheless, recent research shows that its association with venous thromboembolic events is unclear and likely quite weak [46], if not absent [47]. On top of this, homocysteine-lowering treatment was not found to reduce the risk of venous thromboembolism [48]. Given the doubtful role of homocysteinaemia, it is difficult to interpret the role of the vaccine in the pathogenesis of RVO together with homocysteinaemia. It may be inappropriate to base the discussion of the role of the vaccine in RVO on a mildly raised homocysteine level.

### 5.3. Retinal Vasculitis

Another potential new-onset autoimmune condition triggered by COVID-19 vaccines is retinal vasculitis. Ikegami Y et al. and Choi M et al., who noted leaking retinal vessels on patients’ FA images, both suggested that an inflammatory state in retinal vasculature induced by COVID-19 vaccines may be the cause of post-vaccination RVO [5,32]. On the other hand, Nangia P et al. suspected that inflammation-induced thromboembolic events may be the reason behind post-vaccination RVO due to a prompt response to systemic anti-inflammatory therapy [13]. Nangia P et al.’s observation corroborates the hypothesis put forth by Ikegami Y et al. However, vessel leakage on FA is not a specific sign for retinal vasculitis.

Other authors proposed that retinal vasculitis may be involved in retinal vein occlusion. Sarpangala S et al. reported a case of CRVO possibly secondary to retinal vasculitis [49]. Vasculitis following immunisation has been observed for other types of vaccines [50,51], although the causal link is unclear [51]. Thromboembolism in vasculitis has been documented, where interleukin (IL)-1 and IL-6 play an important role in vasculitis-related thrombosis [52]. Aside from retinal vasculitis, the current evidence suggests that mRNA vaccines may bring about transient endothelial dysfunction, particularly after the second dose [53]. Endothelial dysfunction itself, whether vasculitis-related or not, can predispose local vasculature to thrombosis [54].

## 6. How Do We Tell an RVO Is Caused by COVID-19 Vaccination?

Currently, there is no consensus regarding the temporal definition of vaccine-related RVO [55]. Different cut-offs were employed by different authors. From the individual case reports, the interval between symptom onset and vaccination ranged from 15 min to 30 days. In the retrospective case series by Vujosevic S et al., they assumed RVO occurring within 6 weeks to be vaccine-related RVO [33]. Twelve weeks was taken as the cut-off by Hashimoto’s team [41]. We noticed that RVO that occurred some time after vaccination was still considered vaccine related. Nevertheless, the effect of vaccination is questionable in cases where the symptom onset was temporally far from the vaccination. In Hashimoto’s self-controlled case series, the symptom onset of more than two-thirds of cases occurred more than 3 weeks after vaccination [41]. If the temporal definition vaccine-related RVO was set more strictly, the incidence of genuine vaccine-related RVO would be even lower. On the other hand, Feltgen N et al. suggested that there may be an underestimation of vaccine-related retinal vascular events as the pandemic might restrict patients from seeking medical help [55].

The establishment of causality between RVO and vaccination remains difficult, if not impossible, at this stage. It may be tempting to blame the vaccine in cases where none of the risk factors for RVO were present. A concrete conclusion on causality is difficult to make with the scarcity of cases and defects in the design of studies available. None of the studies available have a study design for the determination of causality. Apart from this, we also noticed that various vascular risk factors were present in quite a number of case reports of post-COVID vaccine RVO. Cardiovascular comorbidities were also common among the cases in a retrospective case series (Vujosevic et al.) [33], case–control studies and case-by-case analysis (Feltgen N et al.) [55], and in a matched cohort and self-controlled case series (Hashimoto Y et al.) [41]. In Hashimoto Y et al.’s analyses, where comorbidities were adjusted, no statistically significant difference in incidence rate ratio was found between the vaccinated and non-vaccinated arms. These vascular risk factors may actually be the genuine reason for post-vaccination RVO, in spite of the role of vaccines being unknown.

## 7. Ophthalmic Risks with Future Boosters

Very little is known regarding the effect of the third dose and further doses of COVID-19 vaccines on ophthalmic health. To date, only a few case reports of RVO occurring after a third dose have been found, and no conclusion on the causality between vaccination and RVO can be made [24,37,56]. Newer vaccines, such as the bivalent COVID-19 vaccine, have recently been made available in the market and may be used as boosters for populations susceptible to severe COVID-19 [57]. Data regarding the ophthalmic safety of these newer vaccines is still not comprehensive. Continued efforts in surveillance are needed for investigating ophthalmic side effects of further doses and newer types of COVID-19 vaccine.

There are cases where authors have reported the exacerbation and recurrence of past RVO occurring after COVID-19 vaccination. This has resulted in concerns over the safety of the vaccination in this population, including those who suffered from recent post-COVID-19 vaccination RVO [10]. To the best of our knowledge, only two related cases were reported across the world, and one similar case (anti-hepatitis B vaccines) in the 1990s [24,58]. The causality between vaccines and RVO recurrence or exacerbation can still not be confidently established. Caution in patients with a history of RVO should be practised, but it may not be valid to deter patients with such ophthalmic history from receiving vaccinations. To maximise vaccine safety, the optimisation of the general health condition and proper management of underlying medical illnesses prior to vaccination would be of paramount importance.

## 8. General Discussion

Despite being a sight-threatening condition, post-vaccination RVO, fortunately like other cases of RVO, can be managed effectively with standard treatment. Upon a review of the available evidence, post-vaccination RVO was shown to be a relatively rare ophthalmic adverse event of vaccination. Choi M et al. speculated that COVID-19 vaccines may be the cause of discernibly increased numbers of RVO cases in a short period after vaccination [32]. Such a claim is understandable as there were few cases of RVO that were suspected to be vaccine-related in pre-COVID-19 era. There is, however, insufficient evidence to accuse COVID-19 vaccinations of being one of the potential causes of RVO based on the review of latest evidence. Vaccines may be entirely unrelated to post-vaccination RVO, and the mass vaccination campaigns may have created the illusion of RVO being caused by the vaccines.

In view of the evolution of COVID-19 disease, giving populations further booster doses is gaining popularity worldwide, especially for subjects susceptible to COVID-19. While some studies revealed no statistically significant association between vaccines and venous thromboembolism one month after inoculation with the AstraZeneca vaccine [59,60], there is early evidence showing that the thromboembolic risk of COVID-19 is higher than that of certain vaccines [39,61]. As far as the risk of RVO is concerned, being vaccinated may be safer than being infected. 

## 9. Limitations

This review includes only case reports and studies that were not randomised controlled trials. Causality hence cannot be drawn between RVO and COVID-19 vaccines. The case reports of RVO after COVID-19 vaccination were heterogenous in terms of their reporting style, making direct comparison difficult. Only English articles were included in this review, which may result in bias and an incomplete appraisal of the literature available.

## 10. Conclusions

COVID-19 vaccination has undoubtedly brought the world immense benefits in terms of public health. Though the long-term ophthalmic risk of COVID-19 vaccines is not known, it is believed that the benefits of vaccination outweigh the potential ophthalmic risk significantly. While we are awaiting the long-term side effect profile of COVID-19 vaccines, COVID-19 vaccination should still be advocated with reasonable reassurance for the benefit of population health. At the same time, the ophthalmic community should stay vigilant for any potential vaccine-related adverse impacts on ophthalmic health. 

## Figures and Tables

**Table 1:** A summary of patients’ demographics, comorbidities, presentation, and treatment responses.

Paper	Age/Sex	Vaccine (Dose)	BCVA of the Affected Eye	Interval between Last Dose and Symptom Onset	Comorbidities	Treatment Received	Response to Treatment	Remarks
Ruiz OA et al. [12]	51/F	Moderna (2nd)	8/10	12 days	Hypothyroidism	Not mentioned	VA: 3/10	Concurrent BRAO
Parakh S et al. [9]	31/M	AstraZeneca (1st)	6/9	7 days	Unremarkable	Intravitreal anti-VEGF + folic acid, B6, B12	BCVA 6/6	
Nangia P et al. [13]	13/M	Corbevax	6/7.5	15 days	Unremarkable	Intravenous (IV) pulse methylprednisolone	BCVA 6/6 at 8 months	
Fernández-Vigo JI et al. [14]	69/F	AstraZeneca (1st)	20/100	30 days	Unremarkable	Intravitreal dexamethasone implant	VA: 20/150	Macular atrophy present
Chen Y [7]	72/M	BNT (2nd)	Hand motion	10 days	Unremarkable	Intravitreal aflibercept + intravenous then oral methylprednisolone + pan-retinal photocoagulation	VA: 20/400	Concurrent RAO
Karageorgiou G et al. [15]	60/M	ChAdOx1 (unknown)	20/20	7 days	Obesity	Intravitreal anti-VEGF	Unknown	
Takacs A et al. [10]	35/M	mRNA vaccine (1st)	0.5	14 days	Mild aortic insufficiency, smoker, mild hypertension (139/87 mmHg), lower limb varicosed veins	Aspirin + single dose of intravitreal anti-VEGF	VA: 1.0	
Sodhi PK et al. [10]	43/M	AstraZeneca (1st)	20/630	3 days	Unremarkable	Intravitreal injection of triamcinolone acetonide	VA: 20/200	
Tanaka H et al. [16]	50/F	BNT (1st)	20/25	3 days	Hypertension	Intravitreal ranibizumab	VA: 20/20	
	56/F	BNT (1st)	13/20	3 days	Unremarkable	Intravitreal ranibizumab	VA: 20/20	
Romano D et al. [17]	54/F	AstraZeneca (2nd)	20/400	2 days	Hypertension	Dexamethasone intravitreal implant, laser pan-retinal photocoagulation	VA: 20/200	Ischaemic CRVO
Majumder PD et al. [18]	28/M	AstraZeneca (3rd)	2/60	25 days	Unremarkable	IV pulse and oral steroid	VA: 6/9	
Priluck AZ et al. [19]	57/F	Moderna (2nd)	20/20	3 weeks	Hypertension	Laser, intravitreal anti-VEGF	VA: 20/25	
Sugihara K et al. [20]	38/M	BNT (2nd)	0.9 with myopic correction	2 days	Unremarkable	Intravitreal anti-VEGF	VA: 1.2	
Pur DR et al. [21]	34/M	BNT (1st)	20/20	2 days	Unremarkable	Unknown	VA: 20/20 with residual inferior visual field defect	
Peters MC et al. [22]	71/M	AstraZeneca (1st)	6/60	2 days	Unremarkable	Intravitreal anti-VEGF	Unknown	
Peter MC et al. [22]	58/M	AstraZeneca (1st)	6/18	3 days	Unremarkable	Intravitreal anti-VEGF	Unknown	
	73/F	AstraZeneca (1st)	6/19	3 days	Unremarkable	Intravitreal anti-VEGF	Unknown	
	47/F	Pfizer (1st)	6/9.6	5 days	Hyperthyroidism	Intravitreal anti-VEGF	Unknown	
	36/M	Pfizer (2nd)	6/9	1–3 days	Unremarkable	Intravitreal anti-VEGF	Unknown	
Lee S et al. [11]	34/M	Pfizer (2nd)	Counting finger	10–12 days	Unknown	Intravitreal aflibercept, hyperbaric oxygen, etc.	VA 20/30	Concurrent CRAO
Shah PP et al. [23]	27/F	BNT (2nd)	20/20	26 days	Polycystic ovarian syndrome	Intravitreal anti-VEGF	Significant improvement	
Sonawane NJ et al. [8]	50/M	AstraZeneca (2nd)	6/60	4 days	Diabetes mellitus	Intravitreal anti-VEGF	Unknown	
	43/F	AstraZeneca (2nd)	5/60	3 days	Unremarkable	Close follow-up	Unknown	
Tanaka H et al. [24]	71/F	BNT (2nd)	20/30	1 day	History of BRVO in pre-COVID era, other comorbidities unknown	Intravitreal anti-VEGF	VA: 20/20	
	74/M	BNT (1st)	20/25	1 day	History of BRVO before 1st dose, other comorbidities un-known	Intravitreal anti-VEGF	VA: 20/25	No recurrence detected after 2nd dose
Park HS et al. [25]	68/F	AstraZeneca (1st)	Hand motion	1 day	Dyslipidaemia	Observe	Unknown	
	76/M	Pfizer (1st)	logMAR 0.8	3 days	Hypertension	Observe	Unknown	
	85/F	Pfizer (2nd)	Counting fingers	1 day	Diabetes mellitus, hypertension, old tuberculosis, dementia, end-stage renal disease	Anti-VEGF	Unknown	
	59/M	AstraZeneca (1st)	logMAR 0.8	2 days	Diabetes mellitus, hypertension	Observe	Unknown	
	61/M	AstraZeneca (1st)	logMAR 0.04	2 days	Unremarkable	Anti-VEGF	Unknown	
	79/M	Pfizer (2nd)	logMAR 0.04	3 days	Diabetes mellitus, early gastric cancer	Anti-VEGF	Unknown	
	77/F	Pfizer (1st)	logMAR 0.8	16 days	Hypertension, chronic hepatitis, colon cancer	Anti-VEGF	Unknown	
	63/M	Pfizer (1st)	logMAR 0.01	13 days	Diabetes mellitus	Anti-VEGF	Unknown	
	51/F	AstraZeneca (1st)	logMAR 0.09	21 days	Hypertension	Anti-VEGF	Unknown	
	81/F	Pfizer (1st)	logMAR 0.3	4 days	Hypertension	Observe	Unknown	
	61/M	AstraZeneca (1st)	logMAR 0.9	3 days	Hypertension	Observe	Unknown	
Sacconi E et al. [26]	74/F	Moderna (2nd)	20/40	3 weeks	Atrial fibrillation, breast cancer in remission	Intravitreal anti-VEGF	VA: 20/32	
Ikegami Y et al. [5]	54/F	Moderna (2nd)	No light perception	2 days	Hypothyroidism	Unknown	Unknown	Concurrent CRAO
Endo B et al. [27]	52/M	BNT (1st)	20/20	11 days	Unknown	Intravitreal dexamethasone	Fundal abnormalities improved	
			20/30 [11 days after symptom onset]	N/A	Ditto	Intravitreal anti-VEGF and oral apixaban	VA: 20/20	
Bialasiewicz AA [28]	50/M	BNT (2nd)	0.5	15 min	Atopic dermatitis	low-dose acetylsalicylic acid + monthly intravitreal anti-VEGF injections	VA 1.0	
Goyal M et al. [29]	28/M	Sputnik V	6/9	11 days	Unremarkable	Oral prednisolone and apixaban	VA: 6/6	
Da Silva LSC et al. [30]	66/F	AstraZeneca (unknown)	Unknown	16 days	Dyslipidaemia, increased body-mass index, endometrial hypertrophy	Unknown	Unknown	
	51/M	Pfizer (unknown)	Unknown	6 days	Unknown	Unknown	Unknown	
	66/M	AstraZeneca (unknown)	Unknown	4 days	Hypertension	Unknown	Unknown	
	54/F	AstraZeneca (unknown)	Unknown	10 days	Unknown	Unknown	Unknown	
Girbardt C et al. [31]	81/F	Comirnaty (2nd)	0.05	12 days	Hypertension, primary open angle glaucoma	Intravitreal anti-VEGF	Unknown	Concurrent RAO
Choi M et al. [32]	64/M	AstraZeneca (1st)	20/25 (Snellen)	1 day	Unremarkable	Aspirin	Unknown	
	33/F	BNT (2nd)	20/40 (Snellen)	6 days	Unremarkable	Intravitreal anti-VEGF	Unknown	
	48/M	BNT (3rd)	20/125 (Snellen)	6 days	Unremarkable	Intravitreal anti-VEGF	Unknown	
	69/F	AstraZeneca (1st)	20/20 (Snellen)	3 days	Unremarkable	Aspirin	Unknown	
	66/M	AstraZeneca (2nd)	20/20 (Snellen)	7 days	Unremarkable	Observation	Unknown	
	68/F	AstraZeneca (1st)	Hand motion	1 day	On aspirin for unknown reason, BRVO	Observation	Unknown	
	74/F	AstraZeneca (2nd)	Hand motion	6 days	Hypertension, nasal cavity cancer in remission, on aspirin for unknown reason, BRVO	Intravitreal anti-VEGF, followed by vitrectomy	Unknown	
	63/F	AstraZeneca (1st)	20/630 (same as pre-vaccination)	3 days	CRVO	Intravitreal anti-VEGF	Unknown	
Vujosevic S et al. [33]	69/F	AstraZeneca (1st)	20/32	1 week	Deep vein thrombosis	Laser photocoagulation	VA: 20/20	
	82/F	BNT (2nd)	20/63	2 weeks	Unremarkable	Steroid	VA: 20/40	
	96/F	BNT (2nd)	20/200	1 week	Hypertension, diabetes mellitus	Steroid	VA: 20/200	
	91/F	BNT (2nd)	Counting fingers	1.5 weeks	Unremarkable	Patient refused treatment	VA: Counting fingers	
	78/F	BNT (2nd)	20/25	1 week	Unremarkable	Anti-VEGF	VA: 20/20	
	78/F	BNT (2nd)	20/20	1 week	Unremarkable	None	VA: 20/20	
	70/M	AstraZeneca (1st)	20/20	1 week	Unremarkable	None	VA: 20/20	
	40/M	AstraZeneca (1st)	20/20	2 weeks	Hyperhomocysteinaemia	None	VA: 20/20	
	91/M	BNT (2nd)	20/32	4 weeks	Diabetes mellitus	Steroid	VA: 20/32	
	72/F	BNT (2nd)	20/25	3 weeks	Hypertension, hyperlipidaemia	Steroid	VA: 20/20	
	88/M	BNT (2nd)	20/125	2 weeks	Hypertension, hyperlipidaemia, cardiovascular disease, Alzheimer’s disease, “K prostate”	Steroid	VA: 20/125	
	73/F	AstraZeneca (2nd)	Counting fingers	4 weeks	Hypertension, hyperlipidaemia, cardiovascular disease, neuroendocrine tumour	Steroid	VA: Counting fingers	
	65/F	Jcovden (1st)	20/40	1 week	Hypertension, hyperlipidaemia, diabetes mellitus	Steroid	VA: 20/32	
	72/F	AstraZeneca (1st)	20/40	2 weeks	Hypertension, cardiovascular disease	Steroid	VA: 20/50	

Abbreviations: BCVA: best-corrected visual acuity, BNT: BioNTech vaccine, CRAO: central retinal artery occlusion, CRVO: central retinal vein occlusion, F: female, IV: intravenous, M: male, mmHg: millimetre of mercury, RAO: retinal artery occlusion, RVO: retinal vein occlusion, VA: visual acuity and VEGF: vascular endothelial growth factor.

## Data Availability

Not applicable.

## Supplementary Materials

The following supporting information can be downloaded at: https://www.mdpi.com/article/10.3390/vaccines11081281/s1. Table S1—Patient’s demographics, clinical presentation, vaccination record and ophthalmic findings. Table S2—Patient’s clinical characteristics, treatment received and response to treatment.

## Abbreviation

BNT: BioNTech, BRVO: branch retinal vein occlusion, COVID-19: coronavirus disease 2019; CI: confidence interval, CRAO: central retinal artery occlusion, CRP: C-reactive protein, ESR: erythrocyte sedimentation rate, FA: fluorescein angiogram, IOP: intraocular pressure, OCT: optical coherence tomography, OCTA: optical coherence tomography angiography, RVO: retinal vein occlusion, VITT: vaccine-induced immune thrombocytopenic thrombosis.

## References

[1] Pottegård A., Lund L.C., Karlstad Ø., Dahl J., Andersen M., Hallas J., Lidegaard Ø., Tapia G., Gulseth H.L., Ruiz P.L.D. (2021). Arterial events, venous thromboembolism, thrombocytopenia, and bleeding after vaccination with Oxford-AstraZeneca ChAdOx1-S in Denmark and Norway: Population based cohort study. BMJ.

[2] Berild J.D., Larsen V.B., Thiesson E.M., Lehtonen T., Grøsland M., Helgeland J., Wolhlfahrt J., Hansen J.V., Palmu A.A., Hviid A. (2022). Analysis of thromboembolic and thrombocytopenic events after the AZD1222, BNT162b2, and MRNA-1273 COVID-19 vaccines in 3 Nordic countries. JAMA Netw. Open.

[3] Schulz J.B., Berlit P., Diener H.C., Gerloff C., Greinacher A., Klein C., Petzold G.C., Piccininni M., Poli S., Röhrig R. (2021). COVID-19 vaccine-associated cerebral venous thrombosis in Germany. Ann. Neurol..

[4] World Health Oragnisation (2023). WHO Coronavirus Dashboard. https://covid19.who.int/?mapFilter=vaccinations.

[5] Ikegami Y., Numaga J., Okano N., Fukuda S., Yamamoto H., Terada Y. (2021). Combined central retinal artery and vein occlusion shortly after mRNA-SARS-CoV-2 vaccination. QJM Int. J. Med..

[6] Sodhi P.K., Yadav A., Sharma B., Sharma A., Kumar P. (2022). Central Retinal Vein Occlusion Following the First Dose of COVID Vaccine. Cureus.

[7] Chen Y. (2022). Combined central retinal artery occlusion and vein occlusion with exudative retinal detachment following COVID-19 vaccination. Kaohsiung J. Med. Sci..

[8] Sonawane N., Yadav D., Kota A., Singh H. (2022). Central retinal vein occlusion post-COVID-19 vaccination. Indian J. Ophthalmol..

[9] Parakh S., Maheshwari S., Das S., Vaish H., Luthra G., Agrawal R., Gupta V., Luthra S. (2022). Central Retinal Vein Occlusion Post ChAdOx1 nCoV-19 Vaccination–Can It Be Explained by the Two-hit Hypothesis?. J. Ophthalmic Inflamm. Infect..

[10] Takacs A., Ecsedy M., Nagy Z.Z. (2022). Possible COVID-19 MRNA Vaccine-Induced Case of Unilateral Central Retinal Vein Occlusion. Ocul. Immunol. Inflamm..

[11] Lee S., Sankhala K.K., Bose S., Gallemore R.P. (2022). Combined central retinal artery and vein occlusion with ischemic optic neuropathy after COVID-19 vaccination. Int. Med. Case Rep. J..

[12] Ruiz O.A.G., González-López J.J. (2023). Simultaneous unilateral central retinal vein occlusion and branch retinal artery occlusion after Coronavirus Disease 2019 (COVID-19) mRNA vaccine. Arq. Bras. Oftalmol..

[13] Majumder P.D., Nangia P., Prakash V. (2022). Retinal venous occlusion in a child following Corbevax COVID-19 vaccination. Indian J. Ophthalmol..

[14] Fernández-Vigo J.I., Conde C.P., Burgos-Blasco B., Fernández-Vigo J.A. (2022). Bilateral retinal vein occlusion after two doses of SARS-CoV-2 adenovirus vector-based vaccine. J. Français D’ophtalmologie.

[15] Karageorgiou G., Chronopoulou K., Georgalas I., Kandarakis S., Tservakis I., Petrou P. (2022). Branch retinal vein occlusion following ChAdOx1 nCoV-19 (Oxford-AstraZeneca) vaccine. Eur. J. Ophthalmol..

[16] Tanaka H., Nagasato D., Nakakura S., Nagasawa T., Wakuda H., Kurusu A., Mitamura Y., Tabuchi H. (2022). Branch retinal vein occlusion post severe acute respiratory syndrome coronavirus 2 vaccination. Taiwan J. Ophthalmol..

[17] Romano D., Morescalchi F., Romano V., Semeraro F. (2022). COVID-19 AdenoviralVector Vaccine and Central Retinal Vein Occlusion. Ocul. Immunol. Inflamm..

[18] Majumder P.D., Prakash V. (2022). Retinal venous occlusion following COVID-19 vaccination: Report of a case after third dose and review of the literature. Indian J. Ophthalmol..

[19] Priluck A.Z., Arevalo J.F., Pandit R.R. (2022). Ischemic retinal events after COVID-19 vaccination. Am. J. Ophthalmol. Case Rep..

[20] Sugihara K., Kono M., Tanito M. (2022). Branch retinal vein occlusion after messenger RNA-Based COVID-19 vaccine. Case Rep. Ophthalmol..

[21] Pur D.R., Bursztyn L.L.C.D., Iordanous Y. (2022). Branch retinal vein occlusion in a healthy young man following mRNA COVID-19 vaccination. Am. J. Ophthalmol. Case Rep..

[22] Peters M.C., Cheng S.S.H., Sharma A., Moloney T.P., Franzco S.S.H.C., Franzco A.S., Franzco T.P.M. (2022). Retinal vein occlusion following COVID-19 vaccination. Clin. Exp. Ophthalmol..

[23] Shah P.P., Gelnick S., Jonisch J., Verma R. (2021). Central retinal vein occlusion following BNT162b2 (Pfizer-BioNTech) COVID-19 messenger RNA vaccine. Retin. Cases Brief Rep..

[24] Tanaka H., Nagasato D., Nakakura S., Tanabe H., Nagasawa T., Wakuda H., Imada Y., Mitamura Y., Tabuchi H. (2021). Exacerbation of branch retinal vein occlusion post SARS-CoV2 vaccination: Case reports. Medicine.

[25] Park H.S., Byun Y., Byeon S.H., Kim S.S., Kim Y.J., Lee C.S. (2021). Retinal hemorrhage after SARS-CoV-2 vaccination. J. Clin. Med..

[26] Sacconi R., Simona F., Forte P., Querques G. (2021). Retinal vein occlusion following two doses of mRNA-1237 (moderna) immunization for SARS-CoV-2: A case report. Ophthalmol. Ther..

[27] Endo B., Bahamon S., Martínez-Pulgarín D.F. (2021). Central retinal vein occlusion after mRNA SARS-CoV-2 vaccination: A case report. Indian J. Ophthalmol..

[28] Bialasiewicz A., Farah-Diab M., Mebarki H. (2021). Central retinal vein occlusion occurring immediately after 2nd dose of mRNA SARS-CoV-2 vaccine. Int. Ophthalmol..

[29] Goyal M., Murthy S., Srinivas Y. (2021). Unilateral retinal vein occlusion in a young, healthy male following Sputnik V vaccination. Indian J. Ophthalmol..

[30] Da Silva L.S., Finamor L.P., Andrade G.C., Lima L.H., Zett C., Muccioli C., Sarraf E.P., Marinho P.M., Peruchi J., Oliveira R.D.D.L. (2022). Vascular retinal findings after COVID-19 vaccination in 11 cases: A coincidence or consequence?. Arq. Bras. Oftalmol..

[31] Girbardt C., Busch C., Al-Sheikh M., Gunzinger J.M., Invernizzi A., Xhepa A., Unterlauft J.D., Rehak M. (2021). Retinal vascular events after mRNA and adenoviral-vectored COVID-19 vaccines—A case series. Vaccines.

[32] Choi M., Seo M.-H., Choi K.-E., Lee S., Choi B., Yun C., Kim S.-W., Kim Y.Y. (2022). Vision-Threatening Ocular Adverse Events after Vaccination against Coronavirus Disease 2019. J. Clin. Med..

[33] Vujosevic S., Limoli C., Romano S., Vitale L., Villani E., Nucci P. (2022). Retinal vascular occlusion and SARS-CoV-2 vaccination. Graefe’s Arch. Clin. Exp. Ophthalmol..

[34] Yeo S., Kim H., Lee J., Yi J., Chung Y.-R. (2023). Retinal vascular occlusions in COVID-19 infection and vaccination: A literature review. Graefe’s Arch. Clin. Exp. Ophthalmol..

[35] Su C.K.Y., Au S.C.L. (2022). Isolated and combined unilateral central retinal artery and vein occlusions after vaccination. A review of the literature. J. Stroke Cerebrovasc. Dis..

[36] Yeung M., Su C.K.-Y., Au S.C.L. (2022). Vaccine-related retinal artery occlusion in adults: A review of the current literature. J. Stroke Cerebrovasc. Dis..

[37] Sung S.Y., Jenny L.A., Chang Y.C., Wang N.K., Liu P.K. (2023). Central Retinal Vein Occlusion in a Young Woman with Diabetes and Hypertension after mRNA-Based COVID-19 Vaccination—A Case Report and Brief Review of the Literature. Vaccines.

[38] Li J.-X., Wang Y.-H., Bair H., Hsu S.-B., Chen C., Wei J.C.-C., Lin C.-J. (2023). Risk assessment of retinal vascular occlusion after COVID-19 vaccination. NPJ Vaccines.

[39] Dorney I., Shaia J., Kaelber D.C., Talcott K.E., Singh R.P. (2023). Risk of new retinal vascular occlusion after mRNA COVID-19 vaccination within aggregated electronic health record data. JAMA Ophthalmol..

[40] Jampol L.M., Maguire M.G. (2023). No Red Flags for Risk of Retinal Vascular Occlusion After mRNA COVID-19 Vaccination. JAMA Ophthalmol..

[41] Hashimoto Y., Yamana H., Iwagami M., Ono S., Takeuchi Y., Michihata N., Uemura K., Yasunaga H., Aihara M., Kaburaki T. (2022). Ocular Adverse Events after Coronavirus Disease 2019 mRNA Vaccination: Matched Cohort and Self-Controlled Case Series Studies Using a Large Database. Ophthalmology.

[42] Chen Y., Xu Z., Wang P., Li X.M., Shuai Z.W., Ye D.Q., Pan H.F. (2022). New-onset autoimmune phenomena post-COVID-19 vaccination. Immunology.

[43] Scully M., Singh D., Lown R., Poles A., Solomon T., Levi M., Goldblatt D., Kotoucek P., Thomas W., Lester W. (2021). Pathologic antibodies to platelet factor 4 after ChAdOx1 nCoV-19 vaccination. N. Engl. J. Med..

[44] Klok F.A., Pai M., Huisman M.V., Makris M. (2021). Vaccine-induced immune thrombotic thrombocytopenia. Lancet Haematol..

[45] Morris C.F.M., Tahir M., Arshid S., Castro M.S., Fontes W. (2015). Reconciling the IPC and two-hit models: Dissecting the underlying cellular and molecular mechanisms of two seemingly opposing frameworks. J. Immunol. Res..

[46] Hirmerová J. (2013). Homocysteine and venous thromboembolism—Is there any link?. Cor et Vasa.

[47] Ospina-Romero M., Cannegieter S.C., Heijer M.D., Doggen C.J.M., Rosendaal F.R., Lijfering W.M. (2018). Hyperhomocysteinemia and risk of first venous thrombosis: The influence of (unmeasured) confounding factors. Am. J. Epidemiol..

[48] Ray J.G., Kearon C., Yi Q., Sheridan P., Lonn E., Heart Outcomes Prevention Evaluation 2 (HOPE-2) Investigators (2007). Homocysteine-lowering therapy and risk for venous thromboembolism: A randomized trial. Ann. Intern. Med..

[49] Kamath Y., Sarpangala S., George N., Kulkarni C. (2021). Central retinal vein occlusion secondary to varicella zoster retinal vasculitis in an immunocompetent individual during the COVID-19 pandemic—A case report. Indian J. Ophthalmol..

[50] Watanabe T. (2017). Vasculitis following influenza vaccination: A review of the literature. Curr. Rheumatol. Rev..

[51] Bonetto C., Trotta F., Felicetti P., Alarcón G.S., Santuccio C., Bachtiar N.S., Pernus Y.B., Chandler R., Girolomoni G., Hadden R.D. (2016). Vasculitis as an adverse event following immunization—Systematic literature review. Vaccine.

[52] Springer J., Villa-Forte A. (2013). Thrombosis in vasculitis. Curr. Opin. Rheumatol..

[53] Terentes-Printzios D., Gardikioti V., Solomou E., Emmanouil E., Gourgouli I., Xydis P., Christopoulou G., Georgakopoulos C., Dima I., Miliou A. (2022). The effect of an mRNA vaccine against COVID-19 on endothelial function and arterial stiffness. Hypertens. Res..

[54] Poredos P., Jezovnik M.K. (2017). Endothelial dysfunction and venous thrombosis. Angiology.

[55] Feltgen N., Ach T., Ziemssen F., Quante C.S., Gross O., Abdin A.D., Aisenbrey S., Bartram M.C., Blum M., Brockmann C. (2022). Retinal Vascular Occlusion after COVID-19 Vaccination: More Coincidence than Causal Relationship? Data from a Retrospective Multicentre Study. J. Clin. Med..

[56] Gironi M., D’Aloisio R., Verdina T., Shkurko B., Toto L., Mastropasqua R. (2023). Bilateral Branch Retinal Vein Occlusion after mRNA-SARS-CoV-2 Booster Dose Vaccination. J. Clin. Med..

[57] Offit P.A. (2023). Bivalent COVID-19 Vaccines—A Cautionary Tale. N. Engl. J. Med..

[58] Devin F., Roques G., Disdier P., Rodor F., Weiller P. (1996). Occlusion of central retinal vein after hepatitis B vaccination. Lancet.

[59] Simpson C.R., Shi T., Vasileiou E., Katikireddi S.V., Kerr S., Moore E., McCowan C., Agrawal U., Shah S.A., Ritchie L.D. (2021). First-dose ChAdOx1 and BNT162b2 COVID-19 vaccines and thrombocytopenic, thromboembolic and hemorrhagic events in Scotland. Nat. Med..

[60] Simpson C.R., Kerr S., Katikireddi S.V., McCowan C., Ritchie L.D., Pan J., Stock S.J., Rudan I., Tsang R.S.M., de Lusignan S. (2022). Second-dose ChAdOx1 and BNT162b2 COVID-19 vaccines and thrombocytopenic, thromboembolic and hemorrhagic events in Scotland. Nat. Commun..

[61] Chui C.S.L., Fan M., Wan E.Y.F., Leung M.T.Y., Cheung E., Yan V.K.C., Gao L., Ghebremichael-Weldeselassie Y., Man K.K., Lau K.K. (2022). Thromboembolic events and hemorrhagic stroke after mRNA (BNT162b2) and inactivated (CoronaVac) COVID-19 vaccination: A self-controlled case series study. Eclinicalmedicine.

