# Supplementary material for: Retinal Vein Occlusion after COVID-19 Vaccination—A Review
Source: Vaccines (Basel). 2023 Jul 26;11(8):1281. doi: 10.3390/vaccines11081281 (PMC10459858; doi:10.3390/vaccines11081281)
Supplement: Supplementary file 1 [file vaccines-11-01281-s001.zip › vaccines-2488543-supplementary.pdf]

Retinal vein occlusion after COVID-19 vaccination – A review

Ho-Man Leung [1], Sunny Chi-Lik Au [2]

1. Hospital Authority, Hong Kong
2. Department of Ophthalmology, Tung Wah Eastern Hospital, Hong Kong

SUPPLEMENTARY FILE

Containing 2 tables with references

Table S1 – Patient's demographics, clinical presentation, vaccination record and ophthalmic findings

Paper	Age	Gender	Country	Complaint	Laterality	BCVA of affected eye	Interval between last dose and symptom onset	Dose	Vaccine	Fundal abnormalities of the affected eye	OCT abnormalities of the affected eye	OCTA abnormalities of the affected eye	FA abnormalities of the affected eye
**Ruiz OA et al [1]**	51	F	Spanish	Blurry vision	LE	8/10	12 days	2nd	Moderna	optic disc swelling, diffuse venous tortuosity, retinal whitening along superior temporal arterial branch, widespread dot and blot haemorrhage and peripapillary flame-shaped haemorrhages	retinal edema with disorganization of retinal inner layers (DRIL) within the macula	unknown	occlusion in the superior temporal branch of the retinal artery and a central retinal vein occlusion without cystoid macular edema
**Parakh S et al [2]**	31	M	India	Vision diminution	LE	6/9	7 days	1st	AstraZeneca	dilated tortuous veins, superficial and deep intraretinal haemorrhages in all four quadrants with macular edema, disc hyperemia and few cotton-wool spots	CME and neurosensory detachment, central macular thickness of 485 μm	Flow void areas with reduction in vessel density in the superficial capillary plexus	unknown
**Nangia P et al [3]**	13	M	India	Vision diminution	LE	6/7.5	15 days	1st	Corbevax	blurred disc margin, dilated and tortuous retinal vessels, and scattered flame-shaped hemorrhages in all quadrants	unknown	unknown	delayed venous filling in early phase and blocked fluorescence corresponding to areas of haemorrhage
**Fernández-Vigo JI et al [4]**	69	F	Spain	Asymptomatic - Incidental finding of BRVO on routine F/U	LE	20/20	10 days	1st	AstraZeneca	BRVO changes	no macula edema	non-perfused are next to vein occlusion	
				Sudden visual loss with central scotoma	RE	20/100	30 days	2nd	AstraZeneca	multiple haemorrhages seen	cystoid maculae edema, retinal neurosensorial detachment and outer retina hyperreflectivity	unremarkable (no retinal neovascularisation or remarkable non-perfused regions) at 2 month F/U	unknown

**Chen Y [5]**	72	M	Taiwan	Sudden visual loss	RE	hand motion only	10 days	2nd	BNT	extensive flame-shaped haemorrhages, attenuated retinal arteries and macula whitening	hyperreflectivity of inner retinal layers, loss of retinal structure, subretinal fluid accumulation around macula	unknown	delayed perfusion and extensive non-perfused area, leaking vessels present in the periphery and the disc
**Karageorgiou G et al [6]**	60	M	Greece (Caucasian)	Sudden painless visual loss	RE	20/20	7 days	unknown	Vaxceria (ChAdOx1)	disc swelling, peripapillary intraretinal hemorrhages, dilated tortuous veins, and intraretinal dot-blot, flame-shaped hemorrhages in the upper temporal quadrant.	CME, intraretinal hyperreflectivity, intraretinal CME	unknown	localised hypofluorescence with no late leakage
**Takacs A et al [7]**	35	M	Hungary	1-week onset visual loss	RE	0.5	14 days	1st	mRNA vaccine	haemorrhagic CRVO with papilloedema	cystoid maculae edema, elevated retinal nerve fibre layer (especially superior and inferior parts)	Unknown	unknown
**Sodhi PK et al [8]**	43	M	India	sudden painless vision diminution	LE	20/630	3 days	1st	Covishield	optic disc edema, dilated tortuous veins, dot-blot and flame-shaped haemorrhages across the fundus, and blunted foveal reflex	inner retinal layer disorganisation, disruption of the inner segment-outer segment junction, distorted foveal contour with large cystic spaces, central foveal thickness 854 µm	enlarged distorted foveal avascular zone, capillary non-perfusion seen	delayed venous filling, blocked fluorescence from retinal haemorrhages, capillary non-perfusion areas in the peripheral fundus, no neovascularisation

Paper	Age	Gender	Country	Complaint	Laterality	BCVA of affected eye	Interval between last dose and symptom onset	Dose	Vaccine	Fundal abnormalities of the affected eye	OCT abnormalities of the affected eye	OCTA abnormalities of the affected eye	FA abnormalities of the affected eye
**Tanaka H et al [9]**	50	F	Japan	vision loss	RE	20/25	3 days	1st	BNT	flame-shaped haemorrhages around the temporal superior retinal vein and macular edema	flame-shaped haemorrhages around the temporal superior retinal vein and macular edema	no avascular area found	unknown
	56	F	Japan	visual loss	RE	13/20	3 days	1st	BNT	flame-shaped haemorrhages around the	flame-shaped haemorrhages around the	no avascular area found	unknown

										temporal inferior retinal vein and macular edema	temporal inferior retinal vein and macular edema		
**Romano D et al [10]**	54	F	Italy	painless visual loss	RE	20/400	2 days	2nd	AstraZeneca	disc swelling, retinal haemorrhages in all quadrants, dilated and tortuous retinal veins and altered macular reflex	diffuse macular edema, foveal subretinal fluid	Unknown	delayed arteriovenous transit time, diffuse ischaemia of peripheral retina, retinal haemorrhage, staining of venous wall
**Majumder PD et al [11]**	28	M	India	blurred vision	RE	2/60	25 days	3rd	AstraZeneca/Covishield	disc swelling, dilated and tortuous retinal vessels, flame-shaped and dot-blot haemorrhages, cotton wool spots around posterior pole	macular edema, subretinal fluid, foveal thickness: 823 μm	unknown	early phase: hypofluorescence, delayed venous filling (AV transit time: 42 second), blocked fluorescence corresponding to the areas of haemorrhages
**Priluck AZ et al [12]**	57	F	USA	flashes and floaters	LE	20/20 both OD, OS	3 weeks	2nd	Moderna	superotemporal scattered intraretinal haemorrhages along superior arcade, no macular edema, AV nicking, marked venous tortuosity	unknown	unknown	unknown
**Sugihara K et al [13]**	38	M	Japan	visual diminution	LE	0.9 with myopic correction	2 days	2nd	BNT	retinal haemorrhage and cotton-wool spots (superotemporal region of posterior pole)	subfoveal fluid accumulation, thickened retina in the superior macular region (central foveal thickness 373 μm) in superior macula	unknown	vascular staining and mild macular leakage
**Pur DR et al [14]**	34	M	Canada	blurred vision (inferior visual field), photopsia	RE	OU 20/20	2 days	1st	BNT	superior nasal BRVO (venous dilatation and tortuosity, multiple retinal haemorrhages, cotton wool spots), intraretinal hemoarrhages in the superior retina extending to the periphery	unknown	unknown	delayed venous filling + late staining of affected vessels

**Peters MC et al [15]**	71	M	Australia	visual disturbance	unknown	6/60	2 days	1st	AstraZeneca	unknown	unknown	unknown	unknown
	58	M	Australia	visual disturbance	unknown	6/18	3 days	1st	AstraZeneca	unknown	unknown	unknown	unknown
	73	F	Australia	visual disturbance	unknown	6/19	3 days	1st	AstraZeneca	unknown	unknown	unknown	unknown
	47	F	Australia	visual disturbance	unknown	6/9.6	5 days	1st	Pfizer	unknown	unknown	unknown	unknown
	36	M	Australia	visual disturbance	unknown	6/9	1-3 days	2nd	Pfizer	unknown	unknown	unknown	unknown

Paper	Age	Gender	Country	Complaint	Laterality	BCVA of affected eye	Interval between last dose and symptom onset	Dose	Vaccine	Fundal abnormalities of the affected eye	OCT abnormalities of the affected eye	OCTA abnormalities of the affected eye	FA abnormalities of the affected eye
**Lee S et al [16]**	34	M	(Caucasian)	blurred vision	LE	counting finger	10-12 days	2nd	Pfizer	dilated and tortuous retinal veins, some scattered blot haemorrhages in all quadrants; retinal whitening with a cherry red spot, disc edema looks like NAION (rotation of the edema around the nerve and the progressive edema without CRVO-related progression of the macular edema or blot haemorrhages; superior pole of optic nerve undergoes atrophy after edema resolution also suggests NAION)	exudative macular detachment, mildly thickened retina, no gross cystoid macular edema (baseline RNFL 93 µm OD, 153 µm OS)	unknown	delayed central retinal vein filling, delayed arterial filling (especially distant branches), blot haemorrhage
**Shah PP et al [17]**	27	F	USA (Caucasian)	floaters and wavy lines	LE	OU 20/20	10 days	1st	BNT	peripapillary vessel obscuration, NFL haemorrhage, few dot-and -blot haemorrhage near the macula, tortuous retinal vessel	RNFL and macula thickening without subretinal fluid	unknown	normal AV transit time, no haemorrhage or leakage

				blurred vision	LE	OU 20/20	26 days	2nd	BNT	worsened haemorrhages and maculae edema	new subretinal fluid affecting the fovea and nasal intraretinal fluid	unknown	unknown
**Sonawane NJ et al [18]**	50	M	India	visual diminution	RE	6/60	4 days	2nd	Covishield	edematous disc, dilated and tortuous veins, diffuse retinal haemorrhages, macular edema (LE: mild proliferative diabetic retinopathy changes	CME, central foveal thickness: 571 µm	unknown	unknown
	43	F	India	sudden visual diminution	RE	5/60	3 days	2nd	Covishield	hyperaemic and edematous disc, tortuous veins, and intraretinal hemorrhages in all quadrants	no CME	unknown	patient rejected
**Tanaka H et al [19]**	71	F	Japan	visual loss	LE	20/30	1 day	2nd	BNT	superior temporal BRVO and secondary macular edema	superior temporal BRVO and secondary macular edema	no avascular area found	unknown
	74	M	Japan	visual loss	RE	20/25	1 day	1st	BNT	superior temporal BRVO with secondary macular edema	superior temporal BRVO with secondary macular edema	no avascular area found	unknown

Paper	Age	Gender	Country	Complaint	Laterality	BCVA of affected eye	Interval between last dose and symptom onset	Dose	Vaccine	Fundal abnormalities of the affected eye	OCT abnormalities of the affected eye	OCTA abnormalities of the affected eye	FA abnormalities of the affected eye
**Park HS et al [20]**	68	F	Korea	reduced visual acuity	LE	hand motion	1 day	1st	AstraZeneca	Done, but not available from text	Done, but not available from text	unknown	Done, but not available from text
	76	M	Korea	reduced visual acuity	LE	logMAR 0.8	3 days	1st	Pfizer	Done, but not available from text	Done, but not available from text	unknown	Done, but not available from text
	85	F	Korea	reduced visual acuity	RE	FC10	1 day	2nd	Pfizer	Done, but not available from text	Done, but not available from text	unknown	Done, but not available from text
	59	M	Korea	reduced visual acuity	LE	logMAR 0.8	2 days	1st	AstraZeneca	Done, but not available from text	Done, but not available from text	unknown	Done, but not available from text
	61	M	Korea	reduced visual acuity	RE	logMAR 0.04	2 days	1st	AstraZeneca	Done, but not available from text	Done, but not available from text	unknown	Done, but not available from text

	79	M	Korea	reduced visual acuity	LE	logMAR0.04	2 days	2nd	Pfizer	Done, but not available from text	Done, but not available from text	unknown	Done, but not available from text
	77	F	Korea	reduced visual acuity	LE	logMAR 0.8	16 days	1st	Pfizer	Done, but not available from text	Done, but not available from text	unknown	Done, but not available from text
	63	M	Korea	reduced visual acuity	RE	logMAR 0.01	13 days	1st	Pfizer	Done, but not available from text	Done, but not available from text	unknown	Done, but not available from text
	51	F	Korea	reduced visual acuity	LE	logMAR 0.09	21 days	1st	AstraZeneca	Done, but not available from text	Done, but not available from text	unknown	Done, but not available from text
	81	F	Korea	reduced visual acuity	LE	logMAR 0.3	4 days	1st	Pfizer	Done, but not available from text	Done, but not available from text	unknown	Done, but not available from text
	61	M	Korea	reduced visual acuity	LE	logMAR 0.9	3 days	1st	AstraZeneca	Done, but not available from text	Done, but not available from text	unknown	Done, but not available from text
**Sacconi R et al [21]**	74	F	Italy	painless visual loss	RE	20/32	2 days	2nd	Moderna	venous congestion, extensive blot haemorrhages in inferior quadrants	occluded areas thickened	not done	not done
					RE	declined to 20/40 at 3 week F/U	ditto	ditto	ditto	unknown	week 3: CME with intraretinal fluid; week 5: macula edema extended, significant serous retinal detachment, intraretinal cysts	not done	week 5: delayed venous filling, masking effect from intraretinal haemorrhages and vascular leakage in inferior quadrants
**Ikegami Y et al [22]**	54	F	Japan	sudden painless visual loss	RE	no light perception	2 days	2nd	Moderna	extensive flame-shaped haemorrhages in all quadrants, macula whitening, papilloedema, attenuated retinal arteries	hyper-reflectivity of inner retinal layers, severe thickening of inner retina and loss of organised layered structure of the retina	absence of foveal and perifoveal vascular flow	1 month later: delayed perfusion filling, macula and peripheral retina non-perfused regions, leaky vessels present in all quadrants
**Endo B et al [23]**	52	M	Columbia	sudden blurred vision, esp. nasal and superotemporal quadrants	LE	OU 20/20	15 days	1st	BNT	minimal dot haemorrhages in upper quadrants, dilated tortuous veins in 4 quadrants, disperse exudates [fellow eye: only changes in macular RPE]	no macular edema	unknown	delayed fluorescence in arterial phase; early phase: hypofluorescence near the temporal and inferonasal branches (with blocking areas corresponding to haemorrhages in superior quadrants); late

													phases: temporal branch occlusion given by the absence of venous filling and slight hyperfluorescence in the nasal and temporal potion of CNII
					LE	20/30 (LE) 11 days after symptom onset	/	ditto	ditto	more spot haemorrhages seen	unknown	unknown	unknown

Paper	Age	Gender	Country	Complaint	Laterality	BCVA of affected eye	Interval between last dose and symptom onset	Dose	Vaccine	Fundal abnormalities of the affected eye	OCT abnormalities of the affected eye	OCTA abnormalities of the affected eye	FA abnormalities of the affected eye
**Bialasiewicz AA et al [24]**	50	M	Qatar	retrobulbar pain, red eye, vision reduction	LE	BCVA OU 1.0/0.5	15 minutes	2nd	BNT	unknown	CME, central retinal thickness: 515 my, mild papilledema, intraretinal cysts, subretinal fluid from a mid-peripheral superior leakage area	unknown	unknown
**Goyal M et al [25]**	28	M	India	visual deterioration	RE	6/9, N8	11 days	2nd	Sputnik V	superior hemi-retinal vein occlusion with severe CME	severe macular edema with significant intraretinal and subretinal fluid	unknown	unknown
**Da Silva LSC et al [26]**	66	F	Brazil	/	/	/	16 days	unknown	AstraZeneca	superotemporal intraretinal haemorrhages, macular edema	large central cysts in the inner nuclear layer, smaller perifoveal cysts, subretinal fluids	unknown	unknown
	51	M	Spain	/	/	/	6 days	unknown	Pfizer	diffuse intraretinal haemorrhages emerging from the optic disc, macular edema	large central cysts in the inner nuclear layer, smaller perifoveal cysts, subretinal fluids	unknown	unknown
	66	M	Brazil	/	/	/	4 days	unknown	AstraZeneca	/	/	/	/
	54	F	Brazil	/	/	/	10 days	unknown	AstraZeneca	/	/	/	/
**Girbardt C et al [27]**	81	F	Germany/ Switzerland?	blurred vision	RE	RE: BCVA: 0.05 (decimal);	12 days	2nd	Comirnaty	multiple intraretinal haemorrhages,	hyperreflectivity of inner retinal	unknown	arterial capillary non-perfusion, delayed venous

						LE: 0.25 (decimal)				intact macula (RE); centrally localised haemorrhages, hard exudates, intraretinal edema with exudates in the macula; increased cup-disc ratio (LE)	layers (RE); CME (LE)		filling (RE); vessel leakage inferior to the fovea (LE)
**Choi M et al [28]**	64	M	Korea (Asian)	unknown	RE	20/25 (snellen)	1 day	1st	AstraZeneca	tortuous vessels, scattered blot haemorrhage	/	/	/
	33	F	Korea (Asian)	unknown	RE	20/40 (snellen)	6 days	2nd	BNT	/	/	/	/
	48	M	Korea (Asian)	unknown	RE	20/125 (snellen)	6 days	3rd	BNT	/	/	/	/
	69	F	Korea (Asian)	unknown	LE	20/20 (snellen)	3 days	1st	AstraZeneca	/	/	/	/
	66	M	Korea (Asian)	unknown	LE	20/20 (snellen)	7 days	2nd	AstraZeneca	/	/	/	/
	68	F	Korea (Asian)	unknown	RE	hand motion	1 day	1st	AstraZeneca	/	/	/	/
	74	F	Korea (Asian)	unknown	RE	hand motion	6 days	2nd	AstraZeneca	/	/	/	/
			Korea (Asian)	unknown	LE	hand motion (VA same as pre-vaccination)	ditto	ditto	ditto	/	/	/	/
	63	F	Korea (Asian)	unknown	LE	20/630 (VA same as pre-vaccination)	3 days	1st	AstraZeneca	tortuous vessels with macular edema	/	/	/

Paper	Age	Gender	Country	Complaint	Laterality	BCVA of affected eye	Interval between last dose and symptom onset	Dose	Vaccine	Fundal abnormalities of the affected eye	OCT abnormalities of the affected eye	OCTA abnormalities of the affected eye	FA abnormalities of the affected eye
**Vujosevic S et al [29]**	69	F	Italy	/	RE	20/32	1 week	1st	AstraZeneca	Haemorrhages seen, quadrant iscaehmia	/	/	/
	82	F	Italy	/	RE	20/63	2 weeks	2nd	BNT	Severe edema, haemorrhages seen, quadrant ischaemia	/	/	/
	96	F	Italy	/	RE	20/200	1 week	2nd	BNT	Severe ischaemia with subfoveal neuroretinal detachment,	/	/	/

									haemorrhages seen			
91	F	Italy	/	LE	Counting finger	1.5 weeks	2nd	BNT	Intraocular inflammation, severe edema and haemorrhages	/	/	/
78	F	Italy	/	RE	20/25	1 week	2nd	BNT	Mild edema and haemorrhages only	/	/	/
78	F	Italy	/	LE	20/20	1 week	2nd	BNT	Only mild haemorrhages	/	/	/
70	M	Italy		RE	20/20	1 week	1st	AstraZeneca	Severe haemorrhages	/	/	/
40	M	Italy	/	RE	20/20	2 weeks	1st	AstraZeneca	Mild haemorrhages	/	/	/
91	M	Italy	/	RE	20/32	4 weeks	2nd	BNT	Severe ischaemia with subfoveal neuroretinal detachment, no haemorrhages	/	/	/
72	F	Italy	/	RE	20/25	3 weeks	2nd	BNT	Mild edema and haemorrhages with quadrant ischaemia	/	/	/
88	M	Italy	/	RE	20/125	2 weeks	2nd	BNT	Moderate edema and mild hemorrhages	/	/	/
73	F	Italy	/	RE	Counting fingers	4 weeks	2nd	AstraZeneca	Mild edema with severe haemorrhages	/	/	/
65	F	Italy	/	RE	20/40	1 week	1st	Jcovden	Mild edema, small subfoveal neuroretinal detachment, severe haemorrhages	/	/	/
72	F	Italy	/	LE	20/40	2 weeks	1st	AstraZeneca	Severe haemorrhage with ischaemia in superior quadrants	/	/	/

Table S2 – Patient’s clinical characteristics, treatment received and response to treatment

Paper	Age	Gender	Country	Past medical history	Past ocular history	Laboratory abnormalities	Diagnosis	Management	Response to treatment
**Ruiz OA et al [1]**	51	F	Spanish	hypothyroidism on levothyroxine	unknown	normal	CRVO, BRAO	Not mentioned	BCVA LE dropped to 3/10 at 1 month F/U; No further vascular occlusive episodes and BCVA remained stable at 2 month F/U
**Parakh S et al [2]**	31	M	India	unremarkable, suspected COVID infection shortly before first dose due to significantly high IgG (> 250 U/mL)	unknown	mildly raised serum homocysteine (22.19 µmol/L)	CRVO	Intravitreal anti-VEGF + folic acid, B6, B12	LE BCVA 6/6, DFE and OCTA abnormalities reduced at 8 month F/U, homocysteine level normalised at 11 month F/U
**Nangia P et al [3]**	13	M	India	unremarkable	unknown	unremarkable	CRVO	IV pulse methylprednisolone	BCVA 6/6 after 3 doses of pulse steroid, complete resolution of retinal haemorrhage and disc edema
**Fernández-Vigo JI et al [4]**	69	F	Spain	unremarkable	LE choroidal macular nevus	unknown	BRVO	None given	Fundal abnormalities resolved, fellow eye developed asymptomatic CRVO at 6 week F/U (after 15 days 2nd dose)
				ditto	ditto	unremarkable	CRVO	Intravitreal dexamethasone implant with recurrence of macular edema at 4 months, retreated with intravitreal dexamethasone implant	RE BCVA: 20/150 due to macular atrophy, with complete resolution of macula edema at 2 month F/U - macular edema recurred at 4 month, retreat successfully with intravitreal dexamethasone implant
**Chen Y [5]**	72	M	Taiwan	unremarkable	unknown	normal, except raised d-dimer (547 ng/mL)	CRAO + CRVO + exudative retinal detachment at posterior pole	intravitreal aflibercept + IV then oral methylprednisolone + pan-retinal photocoagulation	RE BCVA: 20/400 at 1 month, OCT showed subsided retinal fluid (exudative retinal detachment) but a disorganised and atrophic retina at 1 month
**Karageorgiou G et al [6]**	60	M	Greece (Caucasian)	unremarkable, apart from obesity (BMI 29.3)	unremarkable, NAD on routine checkup 3 months prior	unremarkable	BRVO	intravitreal anti-VEGF	unknown
**Takacs A et al [7]**	35	M	Hungary	mild aortic insufficiency, smoker, mild hypertension (139/87 mmHg), lower limb varicosed veins	Unknown	slightly reduced prothrombin time, slightly raised antithrombin activity, slightly elevated serum homocysteine, negative	Haemorrhagic CRVO	ASA protect (100 mg daily) + single dose of intravitreal anti-VEGF for RE	RE VA 1.0 with macular edema completely resolved, retinal haemorrhage and papilloedema reduced

						for anti-heparin/PF4 complex (antibody measured 2.5 months after first presentation)			
**Sodhi PK et al [8]**	43	M	India	unremarkable	unknown	unremarkable, except mildly raised HbA1c (6.7%), no risk factors for CRVO, routine blood test grossly normal	CRVO	Intravitreal injection of triamcinolone acetonide	LE BCVA 20/200 and laser performed for NVE and ischaemic areas at 6 month F/U (DFE: haemorrhages and exudates resolved, central foveal thickness: 320 µm

Paper	Age	Gender	Country	Past medical history	Past ocular history	Laboratory abnormalities	Diagnosis	Management	Response to treatment
**Tanaka H et al [9]**	50	F	Japan	hypertension in clinic (147/87 mmHg, breast cancer on tamoxifen	unknown	unknown	BRVO with secondary macular edema	intravitreal ranibizumab	macular edema resolved and BCVA back to 20/20, no recurrence at 2 month
	56	F	Japan	unremarkable	unknown	unknown	BRVO with secondary macular edema	intravitreal ranibizumab	macular edema resolved and BCVA back to 20/20, no recurrence at 2 month
**Romano D et al [10]**	54	F	Italy	arterial hypertension, well-controlled by drugs	unknown	unremarkable	Ischaemic CRVO	0.7 mg dexamethasone intravitreal implant, laser panretinal photocoagulation	BCVA 20/200 on follow-up and macular edema improved
**Majumder PD et al [11]**	28	M	India	unremarkable	unknown	unremarkable	ischaemic CRVO	IV pulse steroid (methylprednisolone), followed by oral steroid (intravitreal anti-VEGF/steroid deferred due to good CME reduction with pulse steroid and the concern regarding sub-foveal migration of hard exudates with sudden CME reduction)	BCVA: 6/9 and most fundal abnormalities resolved at 1 month
**Priluck AZ et al [12]**	57	F	USA	hypertension, drug history: fluticasone, hydrochlorothiazide, fexofenadine	dry eye disease on artificial tears	unknown	superotemporal BRVO	initially offer just observation due to lack of macular edema; 2 months later, intravitreal anti-VEGF given due to drop in vision during conservative treatment	latest BCVA 20/25, OCT showed persistent foveal-sparing macular edema
**Sugihara K et al [13]**	38	M	Japan	unremarkable	unknown	unremarkable (platelet 256 × 10^3/µL, d-dimer < 0.5 µg/mL, protein C 147%, protein S 124%, anticardiolipin-β2-glycoprotein I complex antibody < 0.7 U/mL, anticardiolipin IgG 8.1 U/mL)	BRVO with macula edema	Intravitreal anti-VEGF	LE BCVA: 1.2 since 2 month post-treatment; 7 months post-treatment, fundal abnormalities resolved

**Pur DR et al [14]**	34	M	Canada	unremarkable	laser correction for mild myopia 2 years ago	unremarkable	BRVO	unknown	at 4 month and 7 month F/U: intraretinal haemorrhages resolved with emergence of new macular exudate; at 10 month F/U - BCVA RE: 20/20 with persistent inferior visual field defect
**Peters MC et al [15]**	71	M	Australia	unremarkable	unremarkable	unknown	inferior macula BRVO + CME	Intravitreal anti-VEGF	unknown
	58	M	Australia	unremarkable	ipsilateral pterygium excision	unknown	hemiretinal RVO + CME	Intravitreal anti-VEGF	unknown
	73	F	Australia	hypertension	Fellow eye macula-off rhegmatogenous retinal detachment + macula hole; OU posterior chamber intraocular lens	unknown	inferotemporal BRVO + CME	Intravitreal anti-VEGF	unknown
	47	F	Australia	hyperthyroidism on carbimazole	unremarkable	unknown	superotemporal macula BRVO + CME	Intravitreal anti-VEGF	unknown
	36	M	Australia	unremarkable	unremarkable	unknown	Non-ischaemic CRVO + CME	Intravitreal anti-VEGF	unknown

Paper	Age	Gender	Country	Past medical history	Past ocular history	Laboratory abnormalities	Diagnosis	Management	Response to treatment
**Lee S et al [16]**	34	M	(Caucasian)	unknown	unknown	raised total cholesterol (227), LDL (159), mildly raised ESR (26) [normal CRP]	CRVO + CRAO which evolves into ischaemia optic neuropathy	hyperbaric oxygen; intravitreal aflibercept + (topical) dexamethasone, bromfenac, acetazolamide, trial LMWH – no clinical improvement; high-dose IV methylprednisolone (due to possible NAION) + pentoxifylline + apixaban – BCVA improved to 20/30 3 weeks after first presentation (with arterial and venous circulation restored)	No response to other treatments but BCVA improved to 20/30 after a combination of higher-dose IV methylprednisolone, pentoxifylline and apixaban (with residual inferior altitudinal visual field defect)
**Shah PP et al [17]**	27	F	USA (Caucasian)	PCOS (was on OCP 7 weeks prior to presentation), idiopathic intracranial hypertension, anaemia	unknown	unremarkable	incipient CRVO	Observe	/
				ditto	unknown	unknown	CRVO	intravitreal anti-VEGF	significant improvement in vision and abnormalities on examination in 2 weeks
**Sonawane NJ et al [18]**	50	M	India	poorly controlled DM	unknown	HbA1c: 13.2%, deranged renal function test	CRVO	intravitreal anti-VEGF	unknown
	43	F	India	unknown	unknown	raised ESR, CRP, rheumatoid factor, d-dimer	impending CRVO	close F/U	unknown

**Tanaka H et al [19]**	71	F	Japan	unknown	LE: inferior temporal BRVO (approx. 3.5 years ago) with secondary macular edema of LE, treated with intravitreal anti-VEGF and pathologies resolved with no recurrence on F/U	unknown	superior temporal BRVO and secondary macular edema	intravitreal anti-VEGF	BCVA back to 20/20, macular edema resolved
	74	M	Japan	unknown	RE: superior temporal BRVO without macular edema (around 1.5 years ago), followed up without treatment	unknown	superior temporal BRVO with secondary macular edema	intravitreal anti-VEGF	BCVA still 20/25, no symptom or recurrence detected after his 2nd dose

Paper	Age	Gender	Country	Past medical history	Past ocular history	Laboratory abnormalities	Diagnosis	Management	Response to treatment
**Park HS et al [20]**	68	F	Korea	dyslipidaemia	vitrectomy for vitreous haemorrhage, cataract surgery	not done	RVO	Observe	unknown
	76	M	Korea	hypertension	normal-tension glaucoma, cataract surgery	unremarkable	RVO	Observe	unknown
	85	F	Korea	DM, hypertension, old tuberculosis, dementia, end-stage renal disease	vitrectomy with cataract surgery for vitreous haemorrhage	not done	RVO	anti-VEGF	unknown
	59	M	Korea	DM, hypertension	vitrectomy with secondary IOL, scleral fixation	unremarkable	RVO	Observe	unknown
	61	M	Korea	unremarkable	unremarkable	unremarkable	RVO	anti-VEGF	unknown
	79	M	Korea	DM, early gastric cancer	unremarkable	unremarkable	RVO	anti-VEGF	unknown
	77	F	Korea	hypertension, chronic hepatitis B, colon cancer	cataract surgery	Not done	RVO	anti-VEGF	unknown
	63	M	Korea	DM	diabetic macular edema, intravitreal triamcinolone injection	low haemoglobin and haematocrit	RVO	anti-VEGF	unknown
	51	F	Korea	hypertension	unremarkable	not done	RVO	anti-VEGF	unknown
	81	F	Korea	hypertension	cataract surgery	not done	RVO	Observe	unknown
	61	M	Korea	hypertension	uveitis	not done	RVO	Observe	unknown

**Sacconi R et al [21]**	74	F	Italy	breast cancer in complete remission, atrial fibrillation on drugs with alleged good drug compliance	unknown	not done	hemispheric RVO	None given as patient is already on anticoagulants for AF	/
				ditto	ditto	week 5: platelet 144 × 10^9/L (mildly reduced)	ditto	Intravitreal anti-VEGF only at week 5	BCVA improved to 20/32 and macula edema resolves
**Ikegami Y et al [22]**	54	F	Japan	hypothyroidism	unknown	slightly reduced platelet (129 × 10^9/L)	CRVO + CRAO	Unknown	unknown
**Endo B et al [23]**	52	M	Columbia	unknown	unknown	unremarkable	non-ischaemic CRVO	first given intravitreal dexamethasone	fundus abnormalities improved after 6 days
				unknown	unknown	ditto	ditto	intravitreal anti-VEGF and oral apixaban given due to worsened BCVA and fundal pathologies	BCVA improved to 20/20 and intraretinal haemorrhages reduced after medications

Paper	Age	Gender	Country	Past medical history	Past ocular history	Laboratory abnormalities	Diagnosis	Management	Response to treatment
**Bialasiewicz AA et al [24]**	50	M	Qatar	atopic dermatitis on topicals	unknown	unremarkable	CRVO (haemorrhagic CRVO with ischaemic areas)	low-dose acetylsalicylic acid + monthly intravitreal anti-VEGF injections	BSCVA: 1.0/1.0 + reduction of macular edema (CRT: 319my) 3 days after treatment
**Goyal M et al [25]**	28	M	India	unremarkable ("healthy")	unknown	unremarkable, except markedly raised COVID-19 IgG	HRVO	oral prednisolone in tapering dose and apixaban for 6 weeks	3 days later: BCVA improved to 6/6, N6; marked improvement on OCT and DFE 10 days after treatment
**Da Silva LSC et al [26]**	66	F	Brazil	Raised Apo-A, increased BMI, hysterectomy 4 months ago for endometrial hypertrophy	unknown	unknown	BRVO	unknown	unknown
	51	M	Spain	unknown	unknown	unknown	CRVO	unknown	unknown
	66	M	Brazil	Hypertension	/	/	RVO	unknown	unknown
	54	F	Brazil	/	/	/	RVO	unknown	unknown
**Girbardt C et al [27]**	81	F	Germany/ Switzerland?	Hypertension on drug, under good control	primary open angle glaucoma	Neurological work-up unremarkable, CTA rule out cerebral sinus venous thrombosis	RAO+RVO (RE); BRVO (LE)	intravitreal anti-VEGF for LE	unknown
**Choi M et al [28]**	64	M	Korea (Asian)	unremarkable	unremarkable	unknown	CRVO	observation with aspirin	unknown
	33	F	Korea (Asian)	unremarkable	unremarkable	unknown	CRVO	intravitreal anti-VEGF	unknown
	48	M	Korea (Asian)	unremarkable	unremarkable	unknown	CRVO	intravitreal anti-VEGF	unknown
	69	F	Korea (Asian)	unremarkable	unremarkable	unknown	BRVO	observation with aspirin	unknown
	66	M	Korea (Asian)	unremarkable	unremarkable	unknown	BRVO	observation	unknown
	68	F	Korea (Asian)	unremarkable, on aspirin for unknown reason	BRVO (2020)	unknown	BRVO with vitreous haemorrhage	observation	unknown
	74	F	Korea (Asian)	hypertension, nasal cavity cancer in complete remission, on aspirin for unknown reason	BRVO (2020)	unknown	BRVO with vitreous haemorrhage	intravitreal anti-VEGF followed by vitrectomy as the vitreous haemorrhage did not resolve	unknown

			Korea (Asian)	/	/	/	BRVO with vitreous haemorrhage	intravitreal anti-VEGF followed by vitrectomy as the vitreous haemorrhage did not resolve	unknown
	63	F	Korea (Asian)	/	CRVO (2021)		CRVO decompensation	intravitreal anti-VEGF	unknown

Paper	Age	Gender	Country	Past medical history	Past ocular history	Laboratory abnormalities	Diagnosis	Management	Response to treatment
**Vujosevic S et al [29]**	69	F	Italy	DVT	/	/	Inferotemporal BRVO	Laser photocoagulation	Latest BCVA: 20/20
	82	F	Italy	Unremarkable	/	/	Inferotemporal BRVO	Steroid	Latest BCVA: 20/40
	96	F	Italy	Hypertension, DM	/	/	CRVO	Steroid	Latest BCVA: 20/200
	91	F	Italy	unremarkable	/	/	CRVO	Patient refused treatment	Latest BCVA: Counting finger
	78	F	Italy	unremarkable	/	/	Superotemporal BRVO	Anti-VEGF	Latest BCVA: 20/20
	78	F	Italy	unremarkable	/	/	Superotemporal BRVO	None	Latest BCVA: 20/20
	70	M	Italy	unremarkable	/	/	CRVO	None	Latest BCVA: 20/20
	40	M	Italy	Hyperhomocysteinaemia	/	/	BRVO	None	Latest BCVA: 20/20
	91	M	Italy	DM			BRVO	Steroid	Latest BCVA: 20/32
	72	F	Italy	Hypertension, hyperlipiademia	/	/	Superotemporal BRVO	Steroid	Latest BCVA: 20/20
	88	M	Italy	Hypertension, hyperlipidaemia, cardiovascular disease, Alzheimer's disease, “K prostate”	/	/	HRVO	Steroid	Latest BCVA: 20/125
	73	F	Italy	Hypertension, hyperlipidaemia, cardiovascular disease, neuroendocrine tumour	/	/	CRVO	Steroid	Latest BCVA: Counting fingers
	65	F	Italy	Hypertension, hyperlipidaemia, DM	/	/	CRVO	Steroid	Latest BCVA: 20/32
	72	F	Italy	Hypertension, cardiovascular disease	/	/	HRVO	Steroid	Latest BCVA: 20/50

Abbreviations: AV nicking: arteriovenous nicking, Apo-A: apolipoprotein-A, BCVA: best-corrected visual acuity, BMI: body mass index, BRVO: branch retinal vein occlusion, CME: cystoid macular edema, CN: cranial nerve, CRP: C-reactive protein, CRVO: central retinal vein occlusion, CTA: Computed tomography angiogram, DFE: dilated fundal examination, DM: diabetes mellitus, DVT: deep vein thrombosis, ESR: erythrocyte sedimentation rate, F: female, FA: fluorescein angiogram, F/U: follow-up, HRVO: hemiretinal vein occlusion, IgG: immunoglobulin-G, IOP: intraocular pressure, IV: intravenous, LDL: low-density lipoprotein, LE: left eye, M: male, NAD: no abnormalities detected, NAION: non-arterial ischaemic optic neuropathy, OCT: optical coherence tomography, OCTA: optical coherence tomography angiography, OCP: oral contraceptive pills, PCOS: polycystic ovarian syndrome, RE: right eye, RNFL: retinal nerve fibre layer, RPE: retinal pigmented epithelium, VEGF: vascular endothelial growth factor

References

1. Ruiz, O.A.G. and J.J. González-López, *Simultaneous unilateral central retinal vein occlusion and branch retinal artery occlusion after Coronavirus Disease 2019 (COVID-19) mRNA vaccine.* Arquivos Brasileiros de Oftalmologia, 2022.
2. Parakh, S., et al., *Central Retinal Vein Occlusion Post ChAdOx1 nCoV-19 Vaccination–Can It Be Explained by the Two-hit Hypothesis?* 2022.
3. Nangia, P., V.J. Prakash, and P.D. Majumder, *Retinal venous occlusion in a child following Corbevax COVID-19 vaccination.* Indian Journal of Ophthalmology, 2022. **70**(10): p. 3713-3715.
4. Fernández-Vigo, J.I., et al., *Bilateral retinal vein occlusion after two doses of SARS-CoV-2 adenovirus vector-based vaccine.* Journal Français d'Ophtalmologie, 2022. **45**(9): p. e397-e399.
5. Chen, Y.C., *Combined central retinal artery occlusion and vein occlusion with exudative retinal detachment following COVID-19 vaccination.* The Kaohsiung Journal of Medical Sciences, 2022.
6. Karageorgiou, G., et al., *Branch retinal vein occlusion following ChAdOx1 nCoV-19 (Oxford-AstraZeneca) vaccine.* European Journal of Ophthalmology, 2022: p. 11206721221124651.
7. Takacs, A., M. Ecsedy, and Z.Z. Nagy, *Possible COVID-19 MRNA Vaccine-Induced Case of Unilateral Central Retinal Vein Occlusion.* Ocular Immunology and Inflammation, 2022: p. 1-6.
8. Sodhi, P.K., et al., *Central Retinal Vein Occlusion Following the First Dose of COVID Vaccine.* Cureus, 2022. **14**(6).
9. Tanaka, H., et al., *Branch retinal vein occlusion post severe acute respiratory syndrome coronavirus 2 vaccination.* Taiwan Journal of Ophthalmology, 2022. **12**(2): p. 202.
10. Romano, D., et al., *COVID-19 AdenoviralVector Vaccine and Central Retinal Vein Occlusion.* Ocular Immunology and Inflammation, 2022: p. 1-3.
11. Majumder, P.D. and V.J. Prakash, *Retinal venous occlusion following COVID-19 vaccination: Report of a case after third dose and review of the literature.* Indian Journal of Ophthalmology, 2022. **70**(6): p. 2191.
12. Priluck, A.Z., J.F. Arevalo, and R.R. Pandit, *Ischemic retinal events after COVID-19 vaccination.* American Journal of Ophthalmology Case Reports, 2022. **26**: p. 101540.
13. Sugihara, K., M. Kono, and M. Tanito, *Branch retinal vein occlusion after messenger RNA-Based COVID-19 vaccine.* Case Reports in Ophthalmology, 2022. **13**: p. 28-32.
14. Pur, D.R., L.L.C.D. Bursztyn, and Y. Iordanous, *Branch retinal vein occlusion in a healthy young man following mRNA COVID-19 vaccination.* American Journal of Ophthalmology Case Reports, 2022. **26**: p. 101445.
15. Peters, M.C., et al., *Retinal vein occlusion following COVID-19 vaccination.* Clinical & Experimental Ophthalmology, 2022.
16. Lee, S.S., Kamalesh K; Bose, Swaraj; Gallemore, Ron P, *Combined central retinal artery and vein occlusion with ischemic optic neuropathy after COVID-19 vaccination.* International Medical Case Reports Journal, 2022. **15**: p. 7.
17. Shah, P.P., et al., *Central retinal vein occlusion following BNT162b2 (Pfizer-BioNTech) COVID-19 messenger RNA vaccine.* Retinal Cases and Brief Reports, 2022.
18. Sonawane, N.J., et al., *Central retinal vein occlusion post-COVID-19 vaccination.* Indian journal of ophthalmology, 2022. **70**(1): p. 308.
19. Tanaka, H., et al., *Exacerbation of branch retinal vein occlusion post SARS-CoV2 vaccination.* Medicine, 2021. **100**(50).

20. Park, H.S., et al., *Retinal hemorrhage after SARS-CoV-2 vaccination.* Journal of Clinical Medicine, 2021. **10**(23): p. 5705.
21. Sacconi, R., et al., *Retinal vein occlusion following two doses of mRNA-1237 (moderna) immunization for SARS-cov-2: a case report.* Ophthalmology and Therapy, 2022. **11**(1): p. 453-458.
22. Ikegami, Y., et al., *Combined central retinal artery and vein occlusion shortly after mRNA-SARS-CoV-2 vaccination.* QJM: An International Journal of Medicine, 2021. **114**(12): p. 884-885.
23. Endo, B., S. Bahamon, and D.F. Martínez-Pulgarín, *Central retinal vein occlusion after mRNA SARS-CoV-2 vaccination: A case report.* Indian journal of ophthalmology, 2021. **69**(10): p. 2865.
24. Bialasiewicz, A., M. Farah-Diab, and H. Mebarki, *Central retinal vein occlusion occurring immediately after 2nd dose of mRNA SARS-CoV-2 vaccine.* International Ophthalmology, 2021. **41**(12): p. 3889-3892.
25. Goyal, M., S.I. Murthy, and Y. Srinivas, *Unilateral retinal vein occlusion in a young, healthy male following Sputnik V vaccination.* Indian journal of ophthalmology, 2021. **69**(12): p. 3793.
26. Da Silva, L.S., et al., *Vascular retinal findings after COVID-19 vaccination in 11 cases: a coincidence or consequence?* Arquivos Brasileiros de Oftalmologia, 2022. **85**: p. 158-165.
27. Girbardt, C., et al., *Retinal vascular events after mRNA and adenoviral-vectored COVID-19 vaccines—A case series.* Vaccines, 2021. **9**(11): p. 1349.
28. Choi, M., et al., *Vision-Threatening Ocular Adverse Events after Vaccination against Coronavirus Disease 2019.* Journal of Clinical Medicine, 2022. **11**(12): p. 3318.
29. Vujosevic, S., et al., *Retinal vascular occlusion and SARS-CoV-2 vaccination.* Graefe's Archive for Clinical and Experimental Ophthalmology, 2022: p. 1-10.
